# Cell-free oxidized hemoglobin drives reactive oxygen species production and pro-inflammation in an immature primary rat mixed glial cell culture

**DOI:** 10.1186/s12974-020-02052-4

**Published:** 2021-02-11

**Authors:** Alex Adusei Agyemang, Suvi Vallius Kvist, Nathan Brinkman, Thomas Gentinetta, Miriam Illa, Niklas Ortenlöf, Bo Holmqvist, David Ley, Magnus Gram

**Affiliations:** 1 Lund University, Department of Clinical Sciences Lund, Pediatrics, Lund, Sweden; 2 R&D, CSL Behring, Kankakee, IL USA; 3 Research Bern, CSL Behring, Bern, Switzerland; 4 Fetal i+D Fetal Medicine Research Center, BCNatal-Barcelona Center for Maternal-Fetal and Neonatal Medicine (Hospital Clínic and Hospital Sant Joan de Deu), Institut Clínic de Ginecologia, Obstetricia i Neonatologia, Universitat de Barcelona, Barcelona, Spain; 5 ImaGene-iT AB, Medicon Village, Lund, Sweden

**Keywords:** Intraventricular hemorrhage, Hemoglobin metabolites, Haptoglobin, Hemorrhagic cerebrospinal fluid, Mixed glial cells, Redox

## Abstract

**Background:**

Germinal matrix intraventricular hemorrhage (GM-IVH) is associated with deposition of redox active cell-free hemoglobin (Hb), derived from hemorrhagic cerebrospinal fluid (CSF), in the cerebrum and cerebellum. In a recent study, using a preterm rabbit pup model of IVH, intraventricularly administered haptoglobin (Hp), a cell-free Hb scavenger, partially reversed the damaging effects observed following IVH. Together, this suggests that cell-free Hb is central in the pathophysiology of the injury to the immature brain following GM-IVH. An increased understanding of the causal pathways and metabolites involved in eliciting the damaging response following hemorrhage is essential for the continued development and implementation of neuroprotective treatments of GM-IVH in preterm infant.

**Methods:**

We exposed immature primary rat mixed glial cells to hemorrhagic CSF obtained from preterm human infants with IVH (containing a mixture of Hb-metabolites) or to a range of pure Hb-metabolites, incl. oxidized Hb (mainly metHb with iron in Fe^3+^), oxyHb (mainly Fe^2+^), or low equivalents of heme, with or without co-administration with human Hp (a mixture of isotype 2-2/2-1). Following exposure, cellular response, reactive oxygen species (ROS) generation, secretion and expression of pro-inflammatory cytokines and oxidative markers were evaluated.

**Results:**

Exposure of the glial cells to hemorrhagic CSF as well as oxidized Hb, but not oxyHb, resulted in a significantly increased rate of ROS production that positively correlated with the rate of production of pro-inflammatory and oxidative markers. Congruently, exposure to oxidized Hb caused a disintegration of the polygonal cytoskeletal structure of the glial cells in addition to upregulation of F-actin proteins in microglial cells. Co-administration of Hp partially reversed the damaging response of hemorrhagic CSF and oxidized Hb.

**Conclusion:**

Exposure of mixed glial cells to oxidized Hb initiates a pro-inflammatory and oxidative response with cytoskeletal disintegration. Early administration of Hp, aiming to minimize the spontaneous autoxidation of cell-free oxyHb and liberation of heme, may provide a therapeutic benefit in preterm infant with GM-IVH.

## Background

Germinal matrix intraventricular hemorrhage (GM-IVH) in preterm infants is associated with a high incidence of neurodevelopmental impairment leading to cerebral palsy and intellectual disability [1]. Cerebro-cerebellar deposition of the redox active cell-free hemoglobin (Hb), derived from hemorrhagic cerebrospinal fluid (CSF) in the intraventricular space, has been described to be central in the pathophysiology of brain injury following GM-IVH in preterm infants [2, 3]. Following hemolysis, released cell-free Hb within the intraventricular space is spontaneously autoxidized from oxyHb (Fe^2+^-Hb) to metHb (Fe^3+^-Hb). Further downstream reactions can lead to the formation of highly oxidized ferryl Hb (Fe^4+^-Hb), globin radicals, free heme and heme polymers [4–7]. The subsequent degradation results in the generation of bilirubin, carbon monoxide, and free iron. Cell-free Hb and its metabolites are known to be initiators of cytotoxic, oxidative, pro-inflammatory, and apoptotic events inducing tissue damage [8–12]. Although published data clearly describe the involvement of Hb in the development of brain damage [4–7], a limitation of the studies conducted up to date has been the inability to characterize the importance of the different Hb-metabolites responsible for the observed damage.

We have previously shown that accumulated levels of metHb correlated with levels of tumor necrosis factor alpha (TNFα) protein in intraventricular CSF following preterm IVH [13]. Interestingly, and somewhat contradictory, some studies have actually reported beneficial effects following exposure to cell-free Hb. For instance, Amri et al. [14] report that oxyHb in low concentrations protects cortical astroglial cell cultures by inhibiting oxidative stress and caspase activation following exposure to hydrogen peroxide. The protective effect of oxyHb was linked to its ability to induce the protein kinase A and C signal transduction pathways whilst reducing nuclear factor kappa beta (NFΚB) activation [14]. An increased understanding and appreciation of the role of different Hb-metabolites, as well as triggered causal pathways, is vital in order to develop and implement neuroprotective strategies and deducing a possible therapeutic window for intervention with Hb-metabolite scavengers.

Irrespective of the known importance of paracrine signaling between glial cells in brain abnormalities, previous in vitro studies on cerebral hemorrhage have, to the best of our knowledge, focused on single cell types [15–17]. Although a single cell culture will delineate the specific response of a cell type, it may not be a true reflection of the in vivo setting and hence this needs to be addressed.

In this study, we hypothesize that production of reactive oxygen species (ROS) by Hb-metabolites, is central to the detrimental pathways following preterm IVH. Results show that in vitro exposure of an immature primary rat mixed glial cell culture to oxidized Hb, rather than oxyHb, led to a similar damaging response as exposure to hemorrhagic CSF. Furthermore, the rate of ROS production was found to positively correlate with that of pro-inflammatory and oxidative markers. Congruently, oxidized Hb caused structural disintegration and morphological changes in the mixed glia cells. Interestingly, co-administration with haptoglobin (Hp), a cell-free Hb scavenger, could only partially reverse the damaging response of hemorrhagic CSF and of oxidized Hb.

## Materials and methods

### Cell culture

The use of animals was approved by the Swedish Animal Ethics Committee in Lund. Primary mixed glial cell cultures, comprising microglia, astrocytes, and oligodendrocytes, were prepared from postnatal day 1 Sprague Dawley rats (Janvier, Le Genest-Saint-Isle, France). Briefly, cerebral hemispheres were dissected in ice-cold Hank’s balanced salt solution (HBSS, Thermo Fisher, Waltham, MA, USA) in order to carefully remove the cerebellum, eyes, and meninges. The cerebrum was then cut in two cortices to remove all seen vessels. The cerebrum was subsequently minced, vessels removed and the cell mass transferred to a 15 ml tube containing HBSS and centrifuged at 300×*g* for 5 min at room temperature (RT). The supernatant was removed and pre-heated complete culture medium (Dulbecco’s modified eagle medium, DMEM, with glutamine, 4.5 g L-glucose +10% fetal bovine serum (FBS), 1% penicillin and streptomycin, Thermo Fisher) was added. Using a fire-polished glass pipette, a homogenous cell suspension was obtained by pipetting up and down repeatedly. The cell suspension was then directly filtrated through a 40 μm mesh and resuspended in pre-heated complete culture medium. Cells were then seeded in poly-d-lysine (Sigma Aldrich, Summit drive Burlington, MA, USA) coated multi-well plates, flasks or on cover slips (for immunocytochemistry, ICC, see further description below). The glial culture, as visualized microscopically, was a pure heterogenic mixture, largely made of astrocytes and fewer proportions of microglia and oligodendrocytes.

### Preparation of oxyHb, oxidized Hb, and heme

Human oxyHb was purified as previously described [18] from human blood of healthy subjects. The use of human blood was approved by the ethical committee review board for studies in human subjects at Lund University, Lund, Sweden. Oxidized Hb (containing a mixture of Hb-metabolites with mainly Fe^3+^and some proportion of Fe^4+^, free heme, and iron) was prepared by incubating the purified oxyHb solution at 37 °C for 72 h (as described by Gram et al. [11]). The proportion of oxyHb to metHb (Fe^3+^-Hb) was determined by a spectrophotometric scan coupled with the equation as described by Winterbourn [18]. The purified Hb from human blood was found to contain in proportion 99% oxyHb to 1% oxidized Hb, and the subsequently prepared oxidized Hb solution had in proportion 70% oxidized Hb species to 30% oxyHb. Heme (Ferriprotoporphyrin IX chloride) was purchased from Porphyrin Products Inc. (Logan, UT, USA), and a 10-mM stock solution was prepared using dimethyl sulfoxide (Sigma Aldrich). All Hb solutions were purified from endotoxin contamination using the endotoxin-removing product EndoTrap as described by the manufacturer (Hyglos GmbH, Bernried am Starnberger See, Germany).

### Hemorrhagic CSF from preterm infants with GM-IVH

Hemorrhagic CSF was sampled from preterm infants (gestational age at birth 25–28 weeks) after detection of GM-IVH, by spinal tap or ventricular reservoir puncture according to clinical routine in the neonatal unit at Lund University Hospital, Lund, Sweden. Immediately after sampling, the CSF was centrifuged at 2000×*g*, 20 °C for 10 min, pooled and the proportion of oxyHb and metHb was determined as described above. Samples were stored at − 80 °C until further use, as described below. The sampling was performed following written consent from the parents, and the study was approved by the ethical committee review board for studies in human subjects at Lund University, Lund, Sweden.

### Experimental design

Mixed glial cells were grown in culture medium containing 10% FBS until day 5–7, at what point all experiments were performed. Complete medium was removed and cells were incubated at 37 °C with any of the following component (i) hemorrhagic CSF from human preterm infants with IVH (containing a mixture of Hb-metabolites), (ii) oxyHb, (iii) oxidized Hb, or (iv) heme. The components were substituted in fresh serum-free culture medium, containing DMEM supplemented with 2% of antioxidant-free B-27 supplement (Thermo Fisher), prior to their respective addition to the cells. In addition, co-administration with human Hp (a mixture of isotype 2-2/2-1, CSL Behring, Kankakee, IL, USA) or NFKB inhibitor VI benzoxathiole compound (Abcam, Cambridge, UK) dissolved in DMSO was included in some of the experiments. Fresh serum-free culture medium, supplemented with 2% of antioxidant-free B-27 supplement served as the control condition (referred to as “control”) in all the experiments. Details of exposures and incubations are further described in respective figure legends.

After incubation, cell culture medium was collected (for protein analysis) and cells analyzed (for ROS production) or harvest (for RNA extraction and mRNA expression analysis) as described below.

### Measurement of intracellular ROS formation

ROS were detected by measuring the fluorescence of 2, 7-dichlorofluorescein (DCF), which is derived from the deacetylation and oxidation of the non-fluorescent compound DCFH2-DA, as described by the manufacturer (Abcam, Cambridge, UK). At the end of the exposure period, cells were washed twice with phosphate-buffered saline (PBS) and then incubated with 25 μM DCFH2-DA for 45 min at 37 °C in the dark. Fluorescence was measured with excitation at 483 nm and emission at 535 nm using a fluorescence microplate reader (VICTOR 1420 multilabel plate reader, Perkin Elmer, Waltham, MA, USA).

### RNA isolation and real-time PCR

RNA was isolated from mixed glia cell cultures utilizing the RNeasy Micro Kit (Qiagen, Alden, Germany) with purity and concentration confirmed using a NanoDrop ND-1000 spectrophotometer (Saveen & Werner AB, Limhamn, Sweden). The optical density ratio (at 260 nm/280 nm) of extracted RNA samples was always approximately 2.0. One microgram of RNA was used as a template for reverse transcriptase reactions using the iScript RT kit (Bio-Rad, Hercules, CA, USA). cDNA was mixed with iTaq Universal SYBR Green super mix (Bio-Rad). Amplification was performed as described by the manufacturer (Bio-Rad) for 40 cycles in a CFX Connect thermal cycler (Bio-Rad), and data were analyzed using CFX Maestro Software (Bio-Rad). The 2^−ΔΔCT^ method was used to determine fold expression with normalization to glyceraldehyde 3-phosphate dehydrogenase (GAPDH) expression and control samples from untreated cells. Primers (Primer PCR assay from Bio-Rad) for the following genes, inducible nitric oxide synthase (iNOS), heme oxygenase-1 (HO-1), TNFα, and superoxide dismutase 2 (SOD2), were analyzed.

NFKB Signaling Pathway (PARN-0252D, Qiagen, Maryland, USA) and Apoptotic Pathway (PARN-0122D, Qiagen, Maryland, USA) array were performed on all experimental conditions by using 1 μg of pooled RNA as template for reverse transcriptase reactions using the RT^2^ First Strand Kit (Qiagen). cDNA was mixed with RT^2^ SYBR Green qPCR Master mix (Qiagen). Amplification was performed as described by the manufacturer (Bio-Rad) for 40 cycles in a CFX Connect thermal cycler (Bio-Rad), and data were analyzed using CFX Maestro Software (Bio-Rad). Normalization and determination of fold change expression for the panel of genes was done on the Qiagen Data analysis center platform.

### Immunocytochemistry

Immunolabeling of mixed glia cells was performed to investigate the changes in structural morphology, reactivity, and activity of the glia cell types following exposure to the respective experimental condition as indicated in figure legends.

The ICC protocol was carried accordingly. Following exposure, experimental culture medium was removed from wells. Cells were then washed once with PBS and subsequently fixed for 15 min at RT with 4% paraformaldehyde (PFA, in 0.1 M PBS, pH 7.4). PFA solution was removed, cells were washed 3× with PBS and then permeabilized by incubation with 0.1% Triton X-100 in PBS for 10 min at RT. Following washing 3× with PBS, the cells were blocked with 2% bovine serum albumin (BSA, Sigma Aldrich, diluted in PBS) for 1 h at RT. Incubation with primary antibodies (diluted in 2% BSA in PBS) was performed overnight at + 4 °C. The primary antibodies used were as follows: Iba1 (ionized calcium-binding adaptor molecule 1, diluted 1:500, Wako, Neuss, Germany) and phalloidin (diluted 1:900, Thermo Fisher). Following incubation, cells were washed 3× 10 min with 2% BSA in PBS, followed by incubation with respective secondary antibody for 30 min at RT diluted in 2% BSA in PBS. The secondary antibodies were as follows: anti-rabbit antibody conjugated with Alexa Fluor 647 (AF647, Life Technologies, Eugene, OR, USA) and actin conjugated with Texas Red (Thermo Fisher). Cells were subsequently washed 3× 10 min with PBS followed by nuclear staining, using Hoechst stain (diluted 1:10,000 in PBS, Life Technologies), 5 min at RT. After incubation, cells were further washed 3× with PBS and mounted with mounting media to microscopic slide. Sections were examined and photographed using a Zeiss system (Carl Zeiss microscopy, Thornwood, NY, USA). Representative images were further evaluated using ImageJ and image plates generated with Photoshop (Adobe, San Jose, CA, USA).

### ELISA analysis of cell culture medium

The concentration of chemokine ligand 2 (CCL2), chemokine ligand 5 (CCL5), and lipocalin-2, secreted into the cell culture medium of the mixed glial cells following exposure to the respective experimental conditions (as indicated in the figure legends), were determined using the Quantikine (CCL2, CCL5) and DuoSet (lipocalin-2) ELISA Development Kits (R&D Systems, Minneapolis, Minnesota, USA). The analysis was performed according to the instructions from the manufacturer.

### Hp-Hb binding studies

Binding affinity studies were performed in order to ascertain binding of the cell-free Hb-metabolites in the cell culture experiment to the added Hp. Binding kinetics were performed using amine-reactive biosensors (Pall, ForteBio, Cat#: 18-5092) in an Octet Red 96 (Pall, ForteBio). First, the biosensor tips were equilibrated in water for 10 min. After recording a baseline, Hp was immobilized on biosensors in acetate buffer (pH 5.0) for 600 s. For the association step, either oxyHb or metHb was twofold serially diluted (0, 0.94, 1.88, 3.75, 7.50, 15, and 30 nM). As a control, the binding of Hb in the absence of Hp was measured. Raw data were analyzed by Octet System Data analysis version 9.0 (Pall, ForteBio) and fitted to a global 1:1 kinetic model to determine *k*_on_, *k*_off_, and *K*_*D*_.

### Protein extraction and analysis

Following exposure to oxidized Hb, with or without the NFKB inhibitor VI benzoxathiole compound, mixed glial cells were washed with PBS, scraped, and transferred to a centrifugation tube. Following centrifugation (300×*g*, 6 min), the pellet was resuspended in 500 μl complete Cell Extraction Buffer (Invitrogen, UK), containing a protease inhibitor cocktail (Roche, Mannheim, Germany), incubated 30 min on ice and centrifuged (3000×*g*, 10 min, 4 °C). The supernatant (containing the cytosolic fraction was transferred to a new tube). The pellet was resuspended in 50 μl complete Cell Extraction Buffer (as described above) and incubated for 15 min on ice, followed by centrifugation (14,000×*g*, 30 min, 4 °C). The supernatant (containing the nuclear fraction) was transferred to a new tube.

Protein concentrations were quantified in both the cytosolic and the nuclear fraction using the BCA protein assay kit according to instructions from the manufacturer (Thermo Scientific, Waltham, MA, USA). Absorbance was measured at 550 nm (VICTOR 1420 multilabel plate reader, Perkin Elmer). The cytosolic and nuclear fraction was stored at − 80 °C until further analysis.

### Western blot analysis of NFKB pathway activation

SDS-PAGE was performed with pre-cast stain-free 4–20% gels (Mini-Protean TGX, Bio-Rad). Precision Plus Protein All Blue Prestained Protein Standards were used for size determination of proteins (Bio-Rad). After transfer to polyvinylidene difluoride (PVDF) membranes by electroblotting (Transblot® Turbo, Bio-Rad), membranes were incubated in blocking solution (5% non-fat dry milk (Bio-Rad) in PBS containing 0.05% Tween, PBS-T), followed by a primary rabbit anti-p65 antibody (0.5 μg/ml, Abcam, diluted in 5% non-fat dry milk in PBS-T). Swine anti-rabbit IgG horseradish peroxidase (HRP, Dako, Glostrup, Denmark), diluted 1:1700 in 1% non-fat dry milk in PBS-T, was used as secondary antibody. Signals from HRP-conjugates were detected using Clarity Western ECL Substrate (Bio-Rad). Re-blotting against β-actin (cytosolic fraction) or Lamin B1 (nuclear fraction) were performed by using a primary mouse anti-actin antibody (Abcam, diluted 1:10,000 in 1% non-fat dry milk in PBS-T) or rabbit anti-Lamin B1 antibody (Abcam, diluted 1:10,000 in 5% non-fat dry milk in PBS-T). Goat anti-mouse IgG-Alexa Fluor 488 (Invitrogen, diluted 1:5000 in 1% non-fat dry milk in PBS-T) or swine anti-rabbit IgG-HRP (Dako, diluted 1:1700 in 1% non-fat dry milk in PBS-T) were used as secondary antibodies. Signals from HRP-conjugates were detected using Clarity Western ECL Substrate (Bio-Rad). Membranes and gels were imaged and analyzed using the ChemiDoc™ MP System (Bio-Rad).

### Statistics

Statistical analysis was performed with IBM SPSS Statistics version 25 (Armonk, NY, USA). Results are presented as medians (ranges) and displayed as box plots or bar graphs. Comparisons between unrelated groups were performed with the Mann–Whitney *U* test as appropriate. Comparisons between multiple groups were made using the Kruskal–Wallis test followed by pairwise comparison with significance values adjusted for multiple comparisons. *P* values < 0.05 were considered significant.

## Results

### Cellular response following exposure to hemorrhagic CSF

**Fig. 1:**
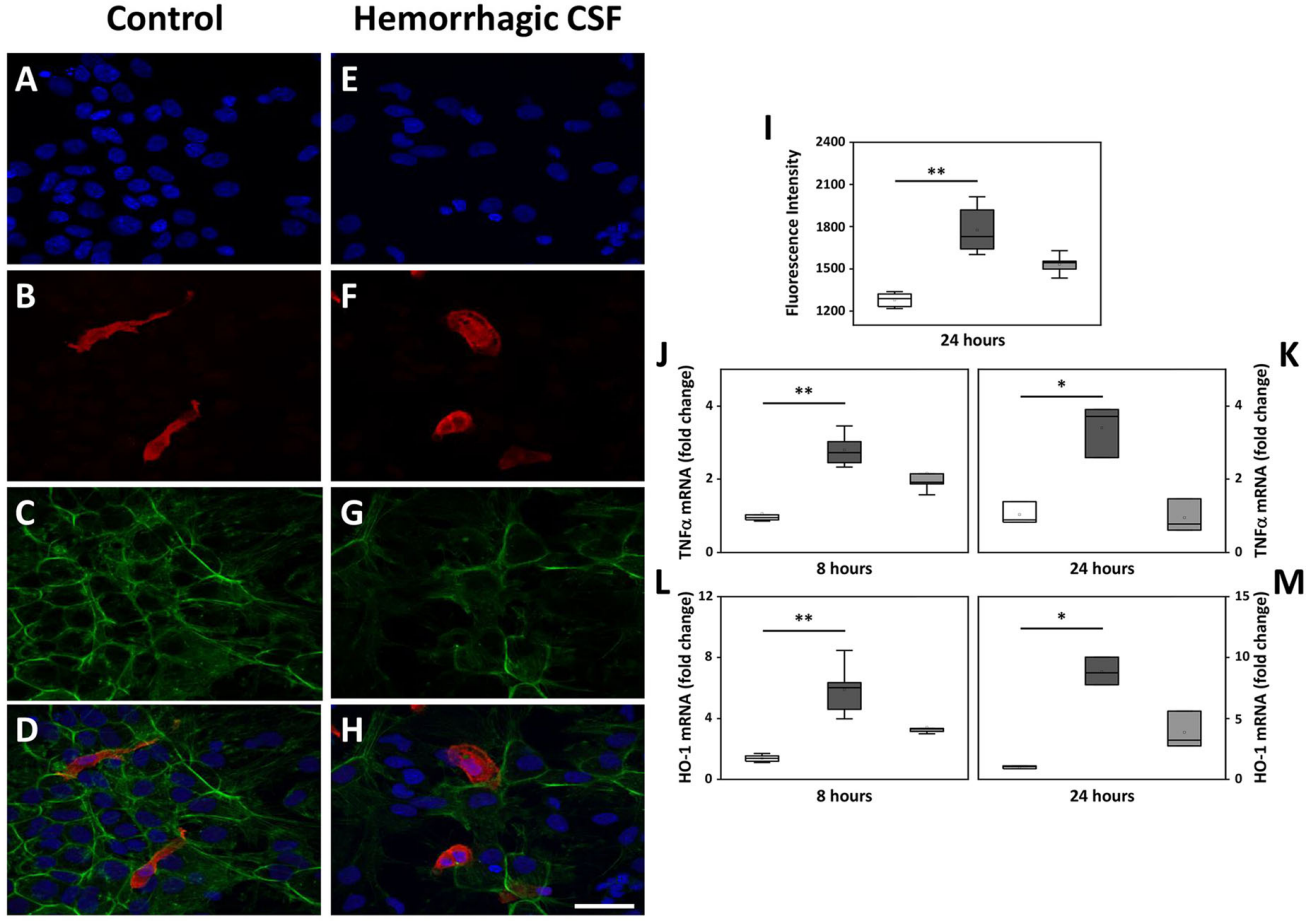
Cellular response following exposure to hemorrhagic CSF. **a**–**h** Representative images are from mixed glial cell cultures following exposure to 3% hemorrhagic CSF (containing 7 μM cell-free Hb) from preterm human infants with GM-IVH. Images illustrate the detected IF labeling of Iba1 (red; **b**, **d**, **f**, **h**), F-actin cytoskeletal protein (phalloidin, green; **c**, **d**, **g**, **h**) and DAPI nuclear staining (blue; **a**, **d**, **e**, **h**), in the wells of mixed glial cells exposed to culture media only (control; **a**–**d**) and in wells exposed to hemorrhagic CSF (**e**–**h**). **d**, **h** Merged images of DAPI, Iba1, and phalloidin. Scale bar in **h** indicates 200 μm and is representative for **a**–**h**. **i** ROS production was investigated (by performing the DCFDA assay as described in materials and method) following a 24 h exposure of mixed glial cells to 3% hemorrhagic CSF (containing 7 µM cell-free Hb). **j-m** TNFα and HO-1 mRNA expression were analyzed at 8 and 24 h after exposure to 3% hemorrhagic CSF (containing 7 µM cell-free Hb). Hemorrhagic CSF (dark gray bars, *n* = 6), co-administration with Hp2-2/2-1 (10 µM, light gray bars, *n* = 6) and control cells (white bars, *n* = 6). Results are presented as box plots displaying medians and 25th and 75th percentiles. Differences between hemorrhagic CSF vs. control and hemorrhagic CSF with co-administered Hp vs. control were analyzed using the Kruskal–Wallis test followed by pairwise comparison with significance values adjusted for multiple comparisons. *P < 0.05, **P < 0.01

Immunofluorescent (IF) labeling of the glial cellular types was evaluated following a 24 h exposure to hemorrhagic CSF and revealed a change in morphology of Iba1 microglia cells from a rod shape (Fig. 1b) to an activated round like flat shape (Fig. 1f). Furthermore, labeled F-actin showed an intermediate change in the polygonal organization of the cytoskeletal structure of the glial cells following exposure to hemorrhagic CSF (Fig. 1g) as compared to control cells (Fig. 1c). These observations were accompanied by a significant induction of ROS production, TNFα and HO-1 mRNA expression (Fig. 1i–m) following exposure to hemorrhagic CSF for 8 and 24 h. Furthermore, the changes were found to be dose-dependent (not shown). Co-administration of Hp, a cell-free Hb scavenger, partially reduced the observed effects (Fig. 1i–m), in some cases down to the levels of control cells (HO-1 at 8 h and TNFα and HO-1 at 24 h).

### Cellular response following exposure to pure Hb-metabolites

**Fig. 2:**
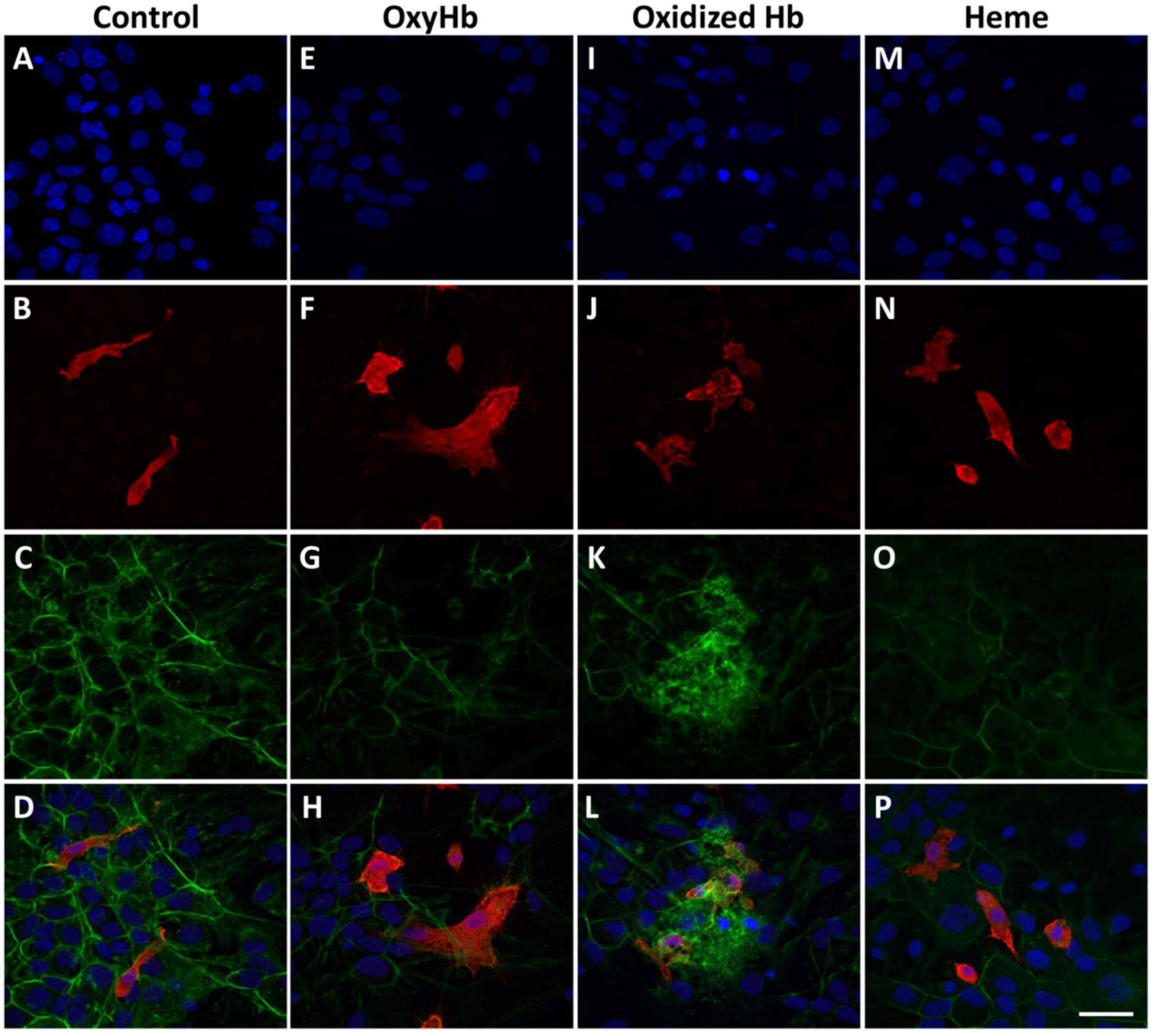
Cellular response following exposure to pure Hb-metabolites. Representative images are from mixed glial cell cultures following exposure to the Hb-metabolites oxyHb (2.5 mg/ml), oxidized Hb (2.5 mg/ml), and heme (15 μM). Images illustrate the detected IF labeling of Iba1 (red; **b**, **d**, **f**, **h**, **j**, **l**, **n**, **p**), F-actin cytoskeletal protein (phalloidin, green; **c**, **d**, **g**, **h**, **k**, **l**, **o**, **p**) and DAPI nuclear staining (blue; **a**, **d**, **e**, **h**, **i**, **l**, **m**, **p**), in the wells of mixed glial cells exposed to culture media only (control; **a**–**d**) and in wells exposed to pure Hb-metabolites (oxyHb 2.5 mg/ml, **e**–**h**; oxidized Hb 2.5 mg/ml, **i**–**l**; and heme 15 μM, **m**–**p**). **d**, **h**, **l**, **p** Merged images of DAPI, Iba1, and phalloidin. Scale bar in **p** indicates 200 μm and is representative for **a**–**p**

**Fig. 3:**
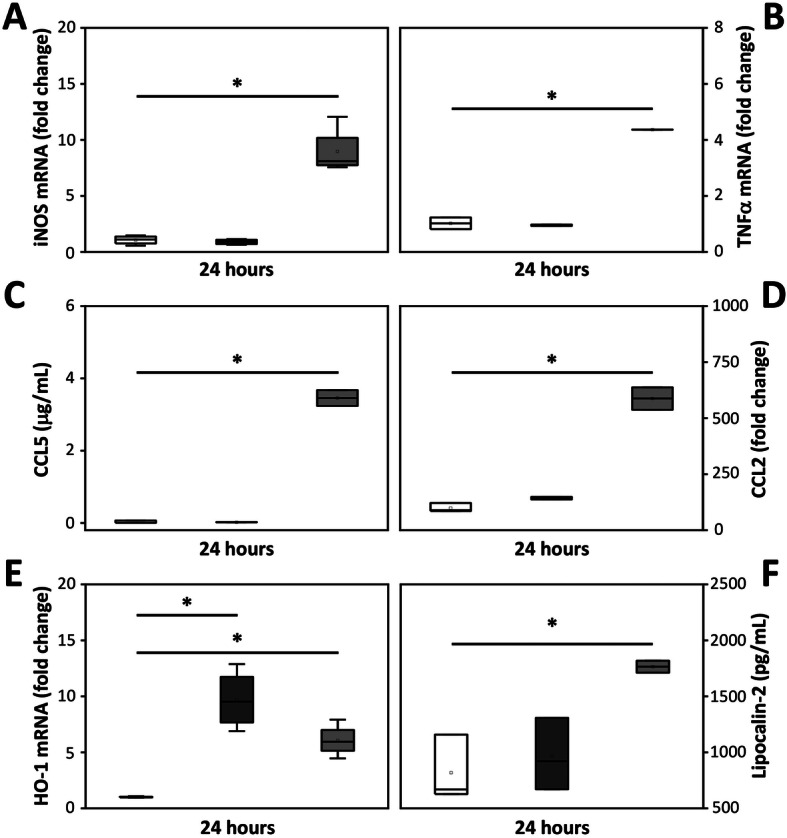
Oxidized Hb induces a pro-inflammatory response. mRNA expression and protein secretion of pro-inflammatory and oxidative stress markers, iNOS (**a**), TNFα (**b**), CCL5 (**c**), CCL2 (**d**), HO-1 (**e**), and lipocalin-2 (**f**) were investigated following exposure of mixed glial cell cultures to pure Hb-metabolites (oxyHb 2.5 mg/ml, dark gray bar, *n* = 4; oxidized Hb 2.5 mg/ml, light gray bar, *n* = 4) or culture medium only (control, white bar, *n* = 4). Results are presented as box plots displaying medians and 25th and 75th percentiles. Differences between oxyHb vs. control, oxidized Hb vs control, and oxidized Hb vs oxyHb were analyzed using the Kruskal–Wallis test followed by pairwise comparison with significance values adjusted for multiple comparisons. *P < 0.05

Iba1 immunoreactivity was investigated to evaluate microglial response following 24 h exposure to pure Hb-metabolites (Fig. 2). All metabolites (oxyHb, oxidized Hb and heme) caused a change in the morphology of microglia cells, from rod shape (control, Fig. 2b) to a round amoeboid like shape (Fig. 2f, j, n). In addition, oxidized Hb, but not oxyHb nor heme, caused a disintegration of the polygonal cytoskeletal structure of the glial cells, as revealed by F-actin labeling (Fig. 2c, g, k, o). Furthermore, an upregulation of F-actin proteins in microglial cells, a marker of microglial metamorphosis, motility, activation, and cytokinesis was observed following exposure to oxidized Hb (Fig. 2k). Congruently, exposure to oxidized Hb but not oxyHb nor heme (heme data not shown) for 24 h caused a significant upregulated mRNA expression of iNOS, TNFα, and HO-1 (Fig. 3a, b, e) as well as an increased protein secretion into the culture medium of CCL5, CCL2, and lipocalin-2 (Fig. 3c, d, f).

### Dose-dependent effect of oxidized Hb

**Fig. 4:**
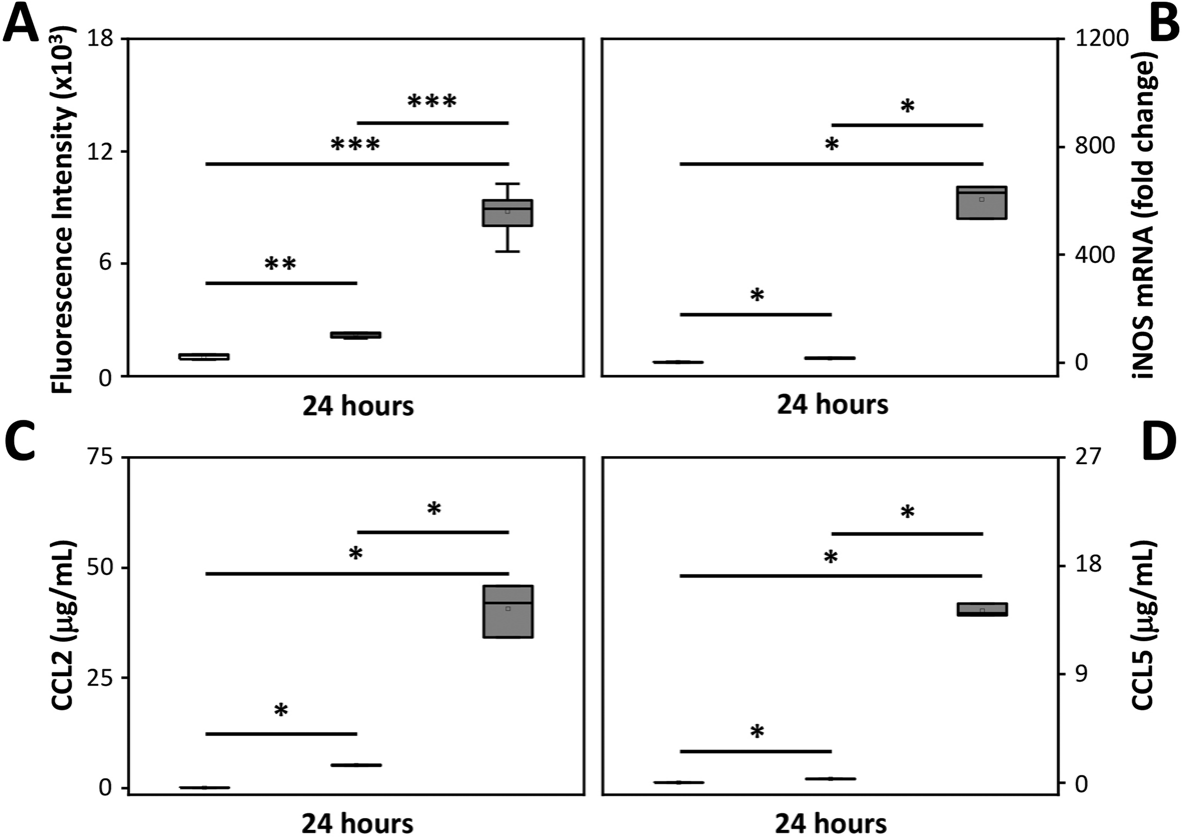
Dose-dependent effect of oxidized Hb. Mixed glial cells were exposed to increasing concentration of oxidized Hb (5 mg/ml, dark grey bars, *n* = 6; 10 mg/ml, light grey bars, *n* = 6) or culture medium only (control, white bar, *n* = 6) for 24 h. Subsequently, ROS production (**a**), mRNA expression of iNOS (**b**) and protein secretion of CCL2 (**c**) and CCL5 (**d**) were analyzed. Results are presented as box plots displaying medians and 25th and 75th percentiles. Differences between 10 mg/ml oxidized Hb vs. control, 5 mg/ml oxidized Hb vs. control, and 10 mg/ml oxidized Hb vs 5 mg/ml oxidized Hb were analyzed using the Kruskal–Wallis test followed by pairwise comparison with significance values adjusted for multiple comparisons. *P < 0.05, **P < 0.01, ***P < 0.001

The mixed glial cell culture was exposed to different concentrations of the oxidized Hb solution for 24 h. Analysis revealed a significant dose-dependent increase of ROS production, mRNA expression of iNOS, and protein secretion of the pro-inflammatory cytokines CCL2 and CCL5 (Fig. 4a–d). In line with above described data, exposure to oxyHb did not result in an increased iNOS mRNA expression nor in an increased secretion of pro-inflammatory cytokines (not shown).

### Effect of Hp on the damage induced by oxidized Hb

**Fig. 5:**
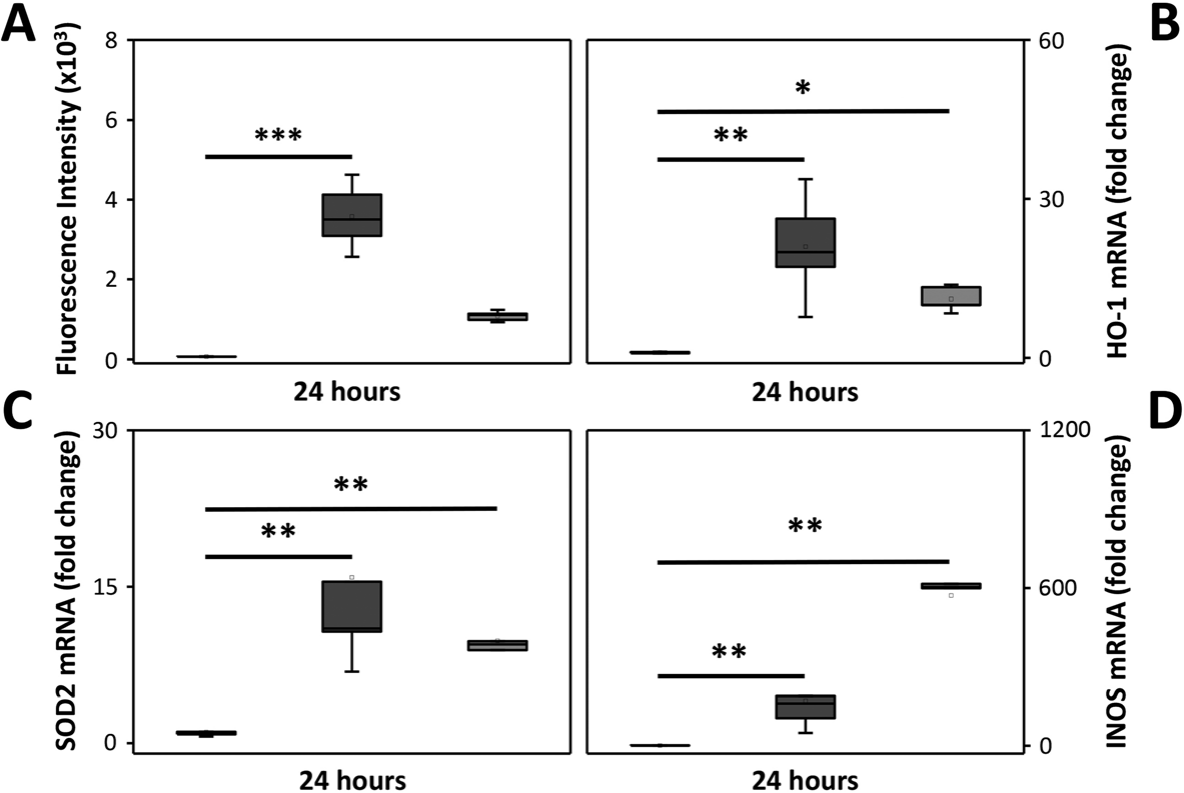
Effect of Hp on the damage induced by oxidized Hb. Mixed glial cells were exposed to oxidized Hb (10 mg/ml, dark grey bars, *n* = 6) with or without co-administration of Hp2-2/2-1 (40 mg/ml, corresponding to a 1:1 molar ratio, light grey bars, *n* = 6) or culture medium only (control, white bars, n = 6) for 24 h. Subsequently, ROS production (**a**), mRNA expression of HO-1 (**b**), SOD2 (**c**), and iNOS (**d**) were analyzed. Results are presented as box plots displaying medians and 25th and 75th percentiles. Differences between oxidized Hb vs. control and oxidized Hb with co-administered Hp vs. control were analyzed using the Kruskal–Wallis test followed by pairwise comparison with significance values adjusted for multiple comparisons. **P < 0.01, ***P < 0.001

The effect of Hp, a cell-free Hb scavenger, to reduce or inhibit the damaging response following exposure of the mixed glial cell culture to oxidized Hb was investigated by co-administration with Hp (in a 1:1 molar ratio, where all cell-free Hb are bound by Hp). A significant reduction on the levels of ROS production, and a tendency towards reduction on the levels of mRNA expression of HO-1 and SOD2 were observed following addition of Hp. Of note, Hp displayed similar effects on lower levels of oxidized Hb, as long as a 1:1 molar ratio or surplus of Hp was added (not shown). Contrarily, no reduction in induced mRNA expression of iNOS by oxidized Hb was observed following co-administration with Hp (Fig. 5a–d).

### Hp binding to Hb-metabolites

To ascertain whether the partially protective effect of Hp, on oxidized Hb-induced response, was due to lack of a strong binding of the oxidized Hb, binding studies were performed with the respective Hb-metabolites. A sensogram showed that the added Hp bound well to both the oxyHb and metHb with a similar binding affinity. The binding curves for the response over time for the different metabolites showed that both oxyHb and metHb bound strongly and irreversibly to the added Hp (Table 1 and Additional file 1 and 2).

**Table 1:** Hb-binding activity of Hp2-2/2-1. Equilibrium constant, association and dissociation constants for the biolayer interferometry experiments. Data expressed as mean ± SD from three independent experiments

Hb-metabolite	***K***_***D*** (**pM**)_	***k***_**on**_ (1/Ms)	***k***_**dis**_ (1/s)
OxyHb	128.7 ± 2.0	2.7 × 10^5^	3.5 × 10^−5^
MetHb	141.4 ± 1.8	2.0 × 10^5^	2.8 × 10^−5^

### NFKB signaling pathway induction by oxidized Hb

**Fig. 6:**
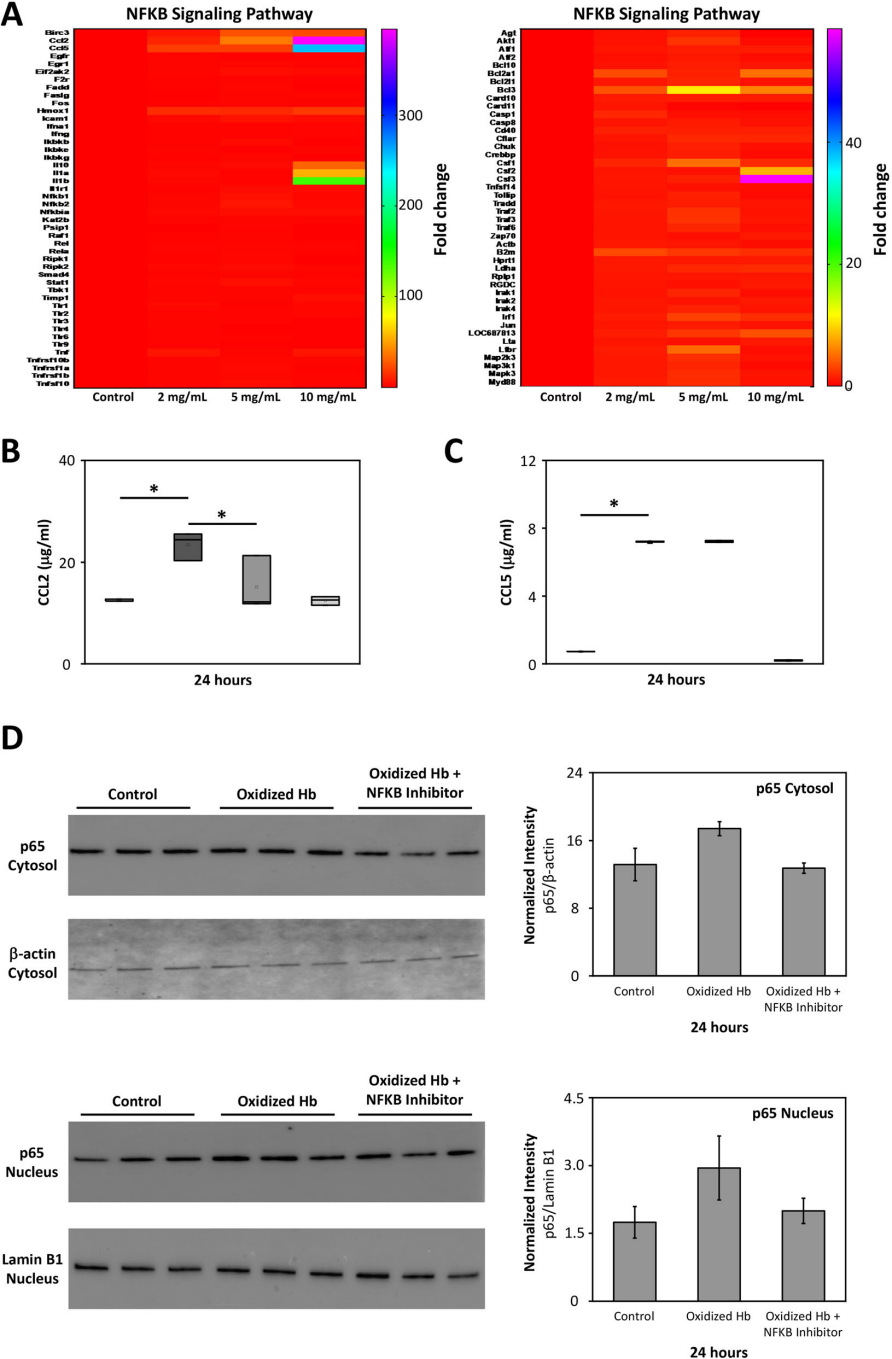
Oxidized Hb induces NFKB signaling pathway. Mixed glial cells were exposed to increasing concentration of oxidized Hb (2–10 mg/ml) or culture medium only (control) for 24 h. **a** A heat map revealing a concentration-dependent upregulation by oxidized Hb in mRNA expression of activators, transcriptional factors, and pro-inflammatory cytokines associated with the NFKB pathway. Addition of the NFKB activation inhibitor VI benzoxathiole (3.0 μM) in the presence of oxidized Hb (2.5 mg/ml, grey bar, *n* = 5) partly inhibited the oxidized Hb (dark grey bar, *n* = 5) induced protein level of CCL2 (**b**), but not CCL5 (**c**). Results of **b** and **c** are presented as box plots displaying medians and 25th and 75th percentiles. Control (white bar, *n = 5*) and NFKB activation inhibitor VI benzoxathiole only (light grey, *n = 5*). Protein levels of the NFKB transcription factor p65 were assessed in the cytosolic and nuclear fraction using western blot. A tendency towards increased level of p65, in both the cytosolic and the nuclear fraction, was observed following exposure to oxidized Hb (2.5 mg/ml). Co-administration of the NFKB inhibitor VI benzoxathiole displayed a trend of reduced p65 protein levels (**d**). β-actin (cytosolic fraction) and Lamin B1 (nuclear fraction) were used as protein loading controls and analyzed on the same material as p65. All samples were applied to one membrane. Membranes are from a representative analysis. Densitometric quantification was performed and mean normalized intensity, normalized for respective β-actin (cytosolic) or Lamin B1 (nuclear) intensity, is presented. Differences between oxidized Hb vs. control, oxidized Hb + NFKB inhibitor vs. control, and oxidized Hb + NFKB inhibitor vs. oxidized Hb were analyzed using the Kruskal–Wallis test followed by pairwise comparison with significance values adjusted for multiple comparisons. *P < 0.05

To evaluate if the NFKB signaling pathway might be involved in the pro-inflammatory response observed following exposure to oxidized Hb, a qPCR array targeting NFKB signaling pathway-related genes were analyzed. Results revealed a concentration-dependent upregulation by oxidized Hb in mRNA levels of activators, transcriptional factors, and pro-inflammatory cytokines associated with the pathway (Fig. 6a). To further evaluate the involvement of the NFKB pathway, mixed glial cells were exposed to oxidized Hb with or without the simultaneous co-administration of the NFKB activation inhibitor VI benzoxathiole compound. Data indicated that blocking the activation of NFKB reduced the pro-inflammatory induction of CCL2 observed following exposure to oxidized Hb (Fig. 6b). However, co-administration of the NFKB inhibitor was not observed to reduce the oxidized Hb-induced CCL5 protein secretion (Fig. 6c). The involvement of the NFKB signaling pathway was additionally investigated by analyzing the protein activity of the NFKB transcription factor p65 in mixed glia cells exposed to oxidized Hb. Western blot analysis showed a tendency towards increased level of p65 following exposure to oxidized Hb, both in the cytosolic and the nuclear compartment, which was partly, although not significantly, reduced following co-administration of the NFKB inhibitor (Fig. 6d).

Interestingly, evaluation of a qPCR array targeting apoptosis pathway-related genes, displayed no change in mRNA expression following exposure to oxidized Hb, not even at very high concentrations (e.g., 10 mg/ml of oxidized Hb, data not shown).

## Discussion

In the present study, we show by exposing mixed glial cells to cell-free Hb-metabolites that oxidized Hb (constituting a mixture of Hb-metabolites with mainly Fe^3+^and some proportion of Fe^4+^, free heme, and iron) rather than oxyHb (Fe^2+^) is responsible for the pro-inflammatory and oxidative response observed following IVH. In agreement with previous reports, exposure to hemorrhagic CSF and oxidized Hb induced cellular activation and pro-inflammatory, oxidative, and cytotoxic pathways [8–13]. Co-administration with Hp partially reversed the cellular damage induced, both by exposure to hemorrhagic CSF from preterm human infants with GM-IVH and pure oxidized Hb, indicating that oxidation of cell-free oxyHb (Fe^2+^) is a critical step in the damaging pathway of IVH.

Glial cells play an important role in brain developmental processes following birth [19–22]. They act as first responders to neurotoxic agents, thereby protecting against neuronal injury [23–25]. Notwithstanding, there is now mounting evidence supporting a crucial role for negative interactions between glial cells in propagating injury in brain abnormalities [26–28].

Cell-free Hb is described to be causally involved in the damaging pathway following IVH [2, 10–12]. It has been suggested that this is caused by affecting the redox activity of the extracellular environment and thereby exposing cells and tissues to oxidative stress [11, 13, 29, 30]. In addition to its redox-related effects, heme, a cell-free Hb-metabolite has been described to act as a damage-associated molecular pattern molecule, triggering the innate immunity and hence causing pro-inflammatory damage to cells [31–33].

The release of Hb, from red blood cells, and subsequent physical accumulation within the brain was traditionally considered to be the cause of damage to the brain during intracerebral hemorrhage and trauma [34–36]. However, increasing evidence suggests that Hb-related neurotoxicity may rather derive from Hb breakdown and the generation of downstream metabolites, and not from mere accumulation of the Hb molecule itself [37, 38]. The accumulation of metHb, an oxidized form of cell-free Hb has been reported in other contexts to be cytotoxic. For instance, Kumar et al. reported that cell-free metHb has been found to drive platelets to apoptosis [39]. In malaria, metHb was found, in the presence of heme, to catalyze the formation of a very cytotoxic heme polymer through a single electron transfer mechanism [40].

Here, we exposed a mixed glial cell culture comprised of astrocytes, microglia, and oligodendrocytes to hemorrhagic CSF, obtained by extraction from preterm human infants with GM-IVH, and to pure Hb-metabolites. The concentrations used in this study were guided by the levels of Hb-metabolites previously observed in clinical CSF samples from human preterm infants with IVH [13]. Hemorrhagic CSF, as well as the respective pure Hb-metabolites, was found to induce an upregulation in mRNA expression of the heme sensitive protein, HO-1. However, oxidized Hb but not oxyHb (Fe^2+^) induced a similar response as hemorrhagic CSF, i.e., a dose-dependent induction of ROS production and pro-inflammatory cytokine mRNA expression and protein secretion, with the rate of ROS production positively correlated with that of pro-inflammatory and oxidative markers.

Congruently, oxidized Hb caused a disintegration of the polygonal cytoskeletal structure of the glial cells possibly reflecting a metabolic inhibition leading to depletion of energy stores, in addition to upregulation of F-actin proteins in microglial cells, a marker of microglia metamorphosis, motility, activation, and cytokinesis.

This finding is consistent with previously reported data, where cell-free Hb deposition in the cerebellar white matter, following IVH in the preterm rabbit pup, was associated with activated microgliosis [2]. The upregulation of actin has been suggested to play a key role in the motility and recruitment of microglia to areas of brain inflammation [41].

It is worth noting that exposure of the mixed glial cells to heme could only be done at a relatively low concentration (15 μM), as compared to oxyHb and oxidized Hb, due to an uncontrollable damaging response by high equivalents of pure heme on the cells.

Although, our understanding of the precise signaling pathways that trigger cell-free Hb-related cell death is still limited, previous studies have hinted on inflammatory necroptosis, apoptosis and/or NFKB modulated inflammation [11, 13, 42].

Here, we show that exposure of the mixed glial cells to oxidized Hb caused an induction of mRNA expression of several NFKB-related genes as well as protein mediators of the NFKB signaling pathway, specifically the NFKB transcription factor p65. Furthermore, addition of the NFKB inhibitor VI benzoxathiole to the mixed glial culture partly abrogated the aforementioned oxidized Hb induction.

Interestingly, oxidized Hb was not found to induce a change in mRNA levels of genes related to the induction of apoptosis pathway. Collectively, this suggests that the observed effects of oxidized Hb on the mixed glial cells may to some extent be accounted for by activation of the NFKB signaling pathway.

Previous studies have reported a protective effect of Hp, a cell-free Hb scavenger, following IVH in a preterm rabbit pup model [2, 11]. In this study, we observed that Hp to some extent blocked the damaging effects on mixed glial cells, following exposure to hemorrhagic CSF and oxidized Hb. This effect was clearly observed by the significantly reduced production of ROS and the oxidative potential of oxidized Hb. However, Hp was not found to reverse the induced mRNA expression of iNOS. This finding may suggest a non-ROS-mediated induction of iNOS mRNA expression by oxidized Hb.

We therefore hypothesized that Hp might bind the oxidized Hb less effectively. However, using binding studies we could show that the added Hp irreversibly binds strongly with similar affinity to both oxyHb and metHb, which shows that inadequate binding between Hp and metHb does not explain the partial lack of protection following exposure to oxidized Hb. This observation is consistent with the findings of the study by Kapralov and colleagues, where the cytotoxic effect of cell-free Hb on macrophages was retained following binding to Hp in a state of severe inflammation [43]. Furthermore, observations made here, in combination with previous studies, may suggest that Hp will exert a protective effect so long as there is an equimolar ratio, or surplus, of Hp to cell-free Hb [44].

In summary, we found that exposing primary immature rat mixed glial cells to hemorrhagic CSF and oxidized Hb-induced cellular activation and pro-inflammatory, oxidative, and cytotoxic pathways. To the best of our knowledge, this work is the first in vitro study where a mixed glial cell culture and not singular cellular types has been exposed to a spectrum of cell-free Hb-metabolites.

In this study, Hp was found to partially reverse the cellular damage induced by hemorrhagic CSF as well as pure oxidized Hb. Collectively, our observations suggest that (i) the conversion of oxyHb (Fe ^2+^) to metHb (Fe^3+^) presents a major step for initiation of the damaging pathway following IVH and (ii) Hp may have a protective effect following IVH by decreasing the rate of the spontaneous autoxidation of cell-free Hb (Hp binding of oxyHb strongly reduces the ability of the ferrous ion to be oxidized) and by preventing the liberation of heme (through scavenging of cell-free Hb). This suggests that treatment with Hp, with the aim of blocking the pro-inflammatory and oxidative effects of cell-free Hb, should be instituted prior to accumulation of oxidized Hb.

## Conclusion

In this study, we show that the conversion of cell-free oxyHb to oxidized Hb is the initiation step for the damage observed following IVH and that the timeline between the conversion from oxyHb (Fe^2+^) to metHb (Fe^3+^) may provide a therapeutic window for implementation of neuroprotective interventions targeting cell-free Hb.

## Supplementary Information

**Additional file 1.** Hb-binding activity of Hp.

**Additional file 2.** Additional File Legends.

## Data Availability

The datasets used and/or analyzed during the current study are available from the corresponding author on reasonable request.

## Abbreviations

BSA: Bovine serum albumin
CCL2: Chemokine (c-c motif) ligand 2
CCL5: Chemokine (c-c motif) ligand 5
CSF: Cerebrospinal fluid
DCF: 2’,7’-dichloro fluorescein
DMEM: Dulbecco’s modified eagle medium
FBS: Fetal bovine serum
GAPDH: Glyceraldehyde-3-phosphate dehydrogenase
GM-IVH: Germinal matrix intraventricular hemorrhage
Hb: Hemoglobin
HBSS: Hank’s balanced salt solution
HO-1: Heme oxygenase 1
Hp: Haptoglobin
Iba1: Ionized calcium-binding adaptor molecule 1
ICC: Immunocytochemistry
iNOS: Inducible nitric oxide synthase
MetHb: Methemoglobin, ferric hemoglobin
NFKB: Nuclear factor kappa beta
OxyHb: Oxyhemoglobin, ferrous hemoglobin
PFA: Paraformaldehyde
PBS: Phosphate-buffer saline
ROS: Reactive oxygen species
SOD2: Superoxide dismutase 2
TNFα: Tumor necrosis factor alpha
