# Supplementary figures and images for: Cell-free oxidized hemoglobin drives reactive oxygen species production and pro-inflammation in an immature primary rat mixed glial cell culture
Source: J Neuroinflammation. 2021 Feb 11;18:42. doi: 10.1186/s12974-020-02052-4 (PMC7879625; doi:10.1186/s12974-020-02052-4)

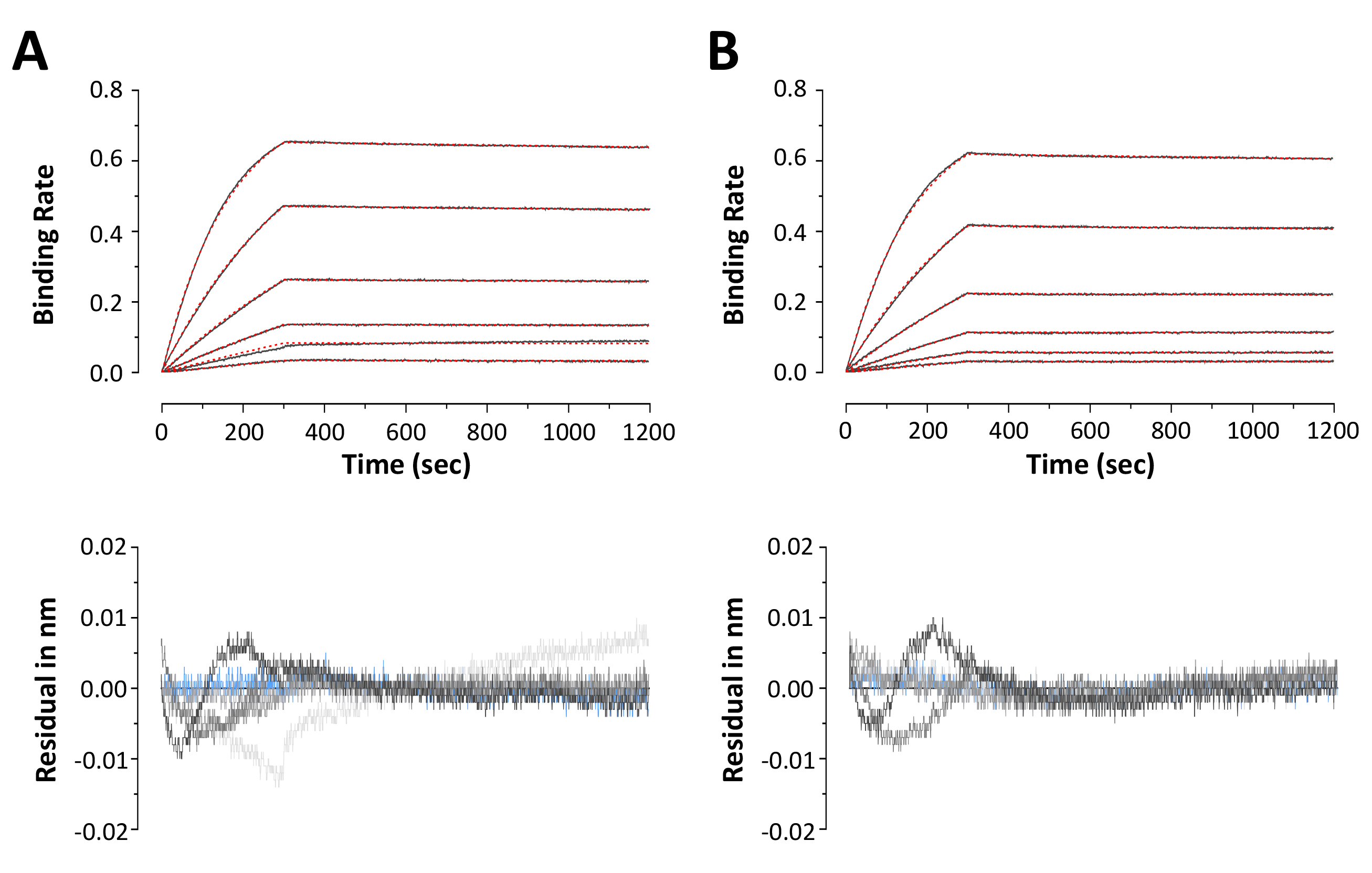

Supplement: Supplementary file 1 — Additional file 1. Hb-binding activity of Hp. [file 12974_2020_2052_MOESM1_ESM.jpg]
