# Supplementary material for: Cell-free oxidized hemoglobin drives reactive oxygen species production and pro-inflammation in an immature primary rat mixed glial cell culture
Source: J Neuroinflammation. 2021 Feb 11;18:42. doi: 10.1186/s12974-020-02052-4 (PMC7879625; doi:10.1186/s12974-020-02052-4)
Supplement: Supplementary file 2 — Additional file 2. Additional File Legends. [file 12974_2020_2052_MOESM2_ESM.docx]

**Additional File Legends**

**Additional File 1. Hb-binding activity of Hp.** Human Hp2-2/2-1 was immobilized on the surface of a biosensor and tested for oxyHb-binding (**a**) and metHb-binding (**b**) by biolayer interferometry. The grey lines represent the association and dissociation curves of a range of Hb concentrations (0, 0.94, 1.88, 3.75, 7.50, 15 and 30 nM). Dashed red lines represent the fitted binding curve according to a global 1:1 kinetic model. The residuals of the fits are plotted below the respective Sensogram. Data shown from one representative experiment for each Hb species.
